# Exosomes from high glucose-treated macrophages promote epithelial–mesenchymal transition of renal tubular epithelial cells via long non-coding RNAs

**DOI:** 10.1186/s12882-023-03065-w

**Published:** 2023-01-30

**Authors:** Huayu Yang, Yu Bai, Chen Fu, Wenhu Liu, Zongli Diao

**Affiliations:** Department of Nephrology, Beijing Friendship Hospital,Capital Medical University, No. 95, Yong’an Road, Xicheng District, 100050 Beijing, PR China

**Keywords:** Macrophages, Exosomes, Long non-coding RNA, High glucose, Renal tubular epithelial cells, Epithelial–mesenchymal transition

## Abstract

**Background:**

Macrophages contribute to epithelial–mesenchymal transition (EMT) in diabetic nephropathy (DN). Exosomal long non-coding RNAs (lncRNAs) derived from macrophages play a major role in transmitting biological information, whereas related studies on DN are rare. Here we investigated the effects of exosomal lncRNAs from high glucose-treated macrophages on EMT.

**Methods:**

High glucose-treated macrophage exosomes (HG-exos) were extracted by coprecipitation and stabilized. Then, mouse renal tubular epithelial cells were treated with HG-exos for 24 h. Expression of E-cadherin, α-smooth muscle actin (α-SMA), and fibronectin was detected by western blotting, qPCR, and immunofluorescence. High-throughput sequencing was then applied to analyze the bioinformatics of HG-exos.

**Results:**

HG-exos inhibited the proliferation of tubular epithelial cells. Additionally, HG-exos markedly upregulated α-SMA and fibronectin expression and downregulated E-cadherin expression in tubular epithelial cells, indicating EMT induction. A total of 378 differentially expressed lncRNAs and 674 differentially expressed mRNAs were identified by high-throughput sequencing of HG-exos. Bioinformatics analysis and subsequent qPCR validation suggested 27 lncRNAs were enriched in the EMT-related MAPK pathway. Among them, ENSMUST00000181751.1, XR_001778608.1, and XR_880236.2 showed high homology with humans.

**Conclusion:**

Exosomes from macrophages induce EMT in DN and lncRNAs in exosomes enriched in the MAPK signaling pathway may be the possible mechanism.

**Supplementary Information:**

The online version contains supplementary material available at 10.1186/s12882-023-03065-w.

## Background

Diabetic nephropathy (DN) is one of the most common complications of diabetes and persistently regarded to be the primary cause of end-stage renal disease [1]. Renal interstitial fibrosis (RIF) is the central pathological pathway of DN and continuous fibrosis eventually leads to renal failure. Epithelial–mesenchymal transition (EMT), a process by which epithelial cells undergo phenotypic conversion, has a critical role in fibrosis development [2].

Accumulation and activation of macrophages in kidney tissue have been found both in DN mice and patients and a reduction of macrophages effectively inhibits RIF progression, indicating the pathological role of macrophages [3–5].

Macrophages may be involved in RIF pathogenesis in DN patients through several mechanisms. Macrophages generate inflammatory cytokines that influence synthesis and degradation of extracellular matrix proteins [6]. Additionally, macrophages have been identified as a major source of myofibroblasts in macrophage–myofibroblast transformation, which subsequently contribute to RIF [7]. Recent reports show that macrophages exert biological effects on recipient cells through a novel type of cell–cell communication mediated by exosomes. Exosomes are membrane-enclosed nanoscale particles that carry bioactive substances including mRNAs and long non-coding RNAs (lncRNAs). Under activation of physiological or pathological factors, exosomes are formed by the intracellular endocytic trafficking pathway [8, 9]. In vitro, high glucose-treated macrophage exosomes (HG-exos) activate glomerular mesangial cells, which induces the proliferation of mesangial cells and secretion of extracellular matrix and inflammatory cytokines [10]. Until now, the role of macrophage-derived exosomes in the mechanism of renal tubular injury in DN has not been reported.

Here, we investigated the role of HG-exos in EMT of renal tubular epithelial cells and differentially expressed lncRNAs in HG-exos to explore the underlying mechanism.

## Methods

### Cell Culture and experimental groups

RAW264.7 macrophages and mouse renal tubular epithelial cells (mRTECs) were purchased from the American Type Culture Collection (Virginia, USA). RAW264.7 macrophages were cultured in DMEM (Gibco, USA) supplemented with 10% fetal bovine serum, penicillin (1 × 10^5^ U/L), and streptomycin (100 mg/L). Low glucose (5.5 mmol/l) DMEM and high glucose (35 mmol/l) DMEM were used. Exosomes from macrophages cultured in low glucose DMEM are referred to as LG-exos.

MRTECs were cultured in EpiCM-a medium (ATCC, USA) supplemented with 10% fetal bovine serum and epidermal growth factor. The culture medium was changed every other day for 7 days. All cells were incubated in a 37 °C in a humidified atmosphere with 5% CO_2_. Cells were passaged with 0.25% trypsin (Sigma, USA) at 70–80% confluence.

Experimental groups included a control group (mRTECs cultured in EpiCM-a medium), LG-exo group (mRTECs cultured with EpiCM-a medium with 100 µg/ml LG-exos), and HG-exo group (mRTECs cultured in EpiCM-a medium with 100 µg/ml HG-exos).

### Isolation and identification of exosomes

Macrophages were incubated in DMEM supplemented with 10% exosome-free FBS and 1% penicillin/streptomycin for 72 h. The culture supernatant as centrifuged at 2000 × *g* for 30 min to remove cells and debris. The sample was mixed with a 0.5 volume of Total Exosome Isolation Reagent (Invitrogen, USA), vortexed, and incubated at 4 °C overnight. Then, the sample was centrifuged at 10,000 × *g* for 1 h. The pellet was then resuspended in PBS.

The ultrastructure of exosome was examined under an Hitachi 7700 transmission electron microscope at 80 kV. The exosome concentration was measured by a ZetaView PMX 110 (Particle Metrix, Germany) under the emission condition of 405 nm. Western blotting was performed to detect expression of exosome-enriched proteins. CD9 and CD81 (Abcam, USA) were used as exosome markers.

### Exosome uptake by MRTECs

Exosomes were resuspended in diluent C solution (200 limulus amebocyte lysate L) and stained with PKH67 (Sigma-Aldrich, USA) for 5 min at room temperature. Then, 0.5% BSA (200 limulus amebocyte lysate L) at the same volume was added to terminate the staining reaction. The PKH6- labeled exosomes were applied to mRTECs for 24 h. After incubation, the cells were washed with PBS and fixed with 4% paraformaldehyde at room temperature. Nuclei were stained with DAPI (Vector Laboratories, USA). Fluorescence was observed under a laser scanning confocal microscope (Olympus, Japan).

MRTECs were also cultured in EpiCM-A supplemented with 10% exosome-free FBS and endothelial growth medium supplement mix at 37 °C with 5% CO_2_. At 70–80% confluence, exosomes or PBS at th**e** same volume was added. Analysis was conducted after 30 min and 24 h.

### Determination of MRTEC viability

MRTEC viability was measured by a cell counting kit-8 assay (Dojindo, Japan, CK-04). At 80% confluence, cells were harvested with 0.25% trypsin and seeded in 96-well plates at a 2 × 10^8^ cells/L. Exosomes were applied in accordance with the experimental groups and five wells were used for each group. After 24 h, the supernatant was discarded and 10 µl CCK-8 solution was added to each well, followed by incubation at 37 °C with 5% CO_2_ for 2 h. The optical density (OD) was measured by an enzyme marker at 450 nm. Cell viability (%) = (OD value of test group-OD value of blank sample) / (OD value of control group-OD value of blank sample) × 100%.

### Western blot analysis

Cells were lysed in RIPA lysis buffer. The protein concentration was measured using a bicinchoninic acid protein assay kit (Thermo Fisher, USA). The supernatant was collected after centrifugation. Protein samples were subjected to sodium dodecyl sulfate polyacrylamide gel electrophoresis. The resolved proteins were transferred to a polyvinylidene difluoride membrane (Merck Millipore, USA). After blocking with 5% BSA, the membrane was incubated with a primary antibody or anti-β-actin antibody as the loading control at 4 °C overnight. The following primary antibodies were used: anti-E-cadherin (1:1000, ab76055, Abcam), anti-α-smooth muscle actin (α-SMA, 1:1000, ab32575, Abcam), anti-fibronectin (1:500, ab2413, Abcam), anti-collagen IV (1:1000; Abcam), anti-plasminogen activator inhibitor-1 (PAI-1, 1:1000, ab241696, Abcam), and anti-β-actin (1:1000, ab8826, Abcam). After washing with TBST, the membrane was incubated at room temperature for 1 h with a secondary antibody. Protein bands were then visualized using hypersensitive ECL. The blots were cut before hybridization with antibodies according to different molecular weight. Band density was analyzed using ImageJ software.

### Quantitative reverse transcription-polymerase chain reaction

Total RNA was extracted from cells and exosomes using TRIzol reagent (Ambion, USA) in accordance with the manufacturer’s instructions. Then, 1 µg RNA was reverse transcribed into cDNA by a Reverse Transcription System (Roche, USA). NCBI Primer BLAST (www.ncbi.NLM.NIH.Gov/tools/primerblast/) and Primer bank (https://pga.mgh.harvard.edu/primerbank/) were used to design primers. RT-qPCR analysis was carried out using a lncRNA qPCR Detection Kit (Tiangen, China) and Fast SYBR Green Master Mix (Thermo Fisher). β-Actin were used as an internal reference. Relative mRNA levels were calculated by the 2^−ΔΔCT^ method. Details of RNA primers are shown in Table 1.

**Table 1:** Primer sequences for quantitative real-time polymerase chain reaction

Gene	Forward (5’ -> 3’)	Reverse (5’ -> 3’)
Collagen IV	CTGGCACAAAAGGGACGAG	ACGTGGCCGAGAATTTCACC
α-SMA	GTCCCAGACATCAGGGAGTAA	TCGGATACTTCAGCGTCAGGA
E-cadherin	CAGGTCTCCTCATGGCTTTGC	CTTCCGAAAAGAAGGCTGTCC
fibronectin	GCTCAGCAAATCGTGCAGC	CTAGGTAGGTCCGTTCCCACT
ENSMUST00000181751.1	AGCTACACCTTCTTCTTGGACTG	ACCAAGCTGTACCAGAGTGC
XR_001778608.1	TTTTCAGCTAGAGCACCCCC	ATGAGAAGGGCAGTCTGGGA
XR_880236.2	GTCTGATGGGGTCAGTGCAT	TTGGGGACTGTGTAATCGGG
β-actin	GGCTGTATTCCCCTCCATCG	CCAGTTGGTAACAATGCCATGT

Abbreviation: α-SMA:α-smooth muscle actin

### Immunofluorescence staining

Cells were fixed with 4% paraformaldehyde for 10 min. Then, the cells were permeabilized with 0.5% Triton X-100 (Sigma) for 10 min, followed by blocking with 5% BSA at room temperature for 1 h. Primary antibodies against α-SMA (1:500, ab32575, Abcam), E-cadherin (1ug/ml, ab76055, Abcam), and fibronectin (1:500, ab2413, Abcam) were applied at 4 °C overnight. After washing with PBS three times, the cells were incubated with fluorescent dye-labeled secondary antibodies in the dark for 1 h at room temperature and then with 0.1% DAPI for 10 min. The cells were observed under an inverted fluorescence microscope and photographed.

### RNA sequencing

To identify differentially expressed mRNAs (DEmRNAs) and differentially expressed lncRNAs (DElncRNAs) in macrophage exosomes after high glucose stimulation, we performed high-throughput sequencing. The culture supernatant was mixed with Ribo™ Exosome Isolation Reagent (Ribobio, China) and exosome isolation was performed. Exosomal RNA was extracted by Magzol Reagent (Magen, China). Then, a NEBNext® Ultra™ RNA Library Prep Kit was used to construct RNA-seq libraries. Sequencing was conducted using the Illumina platform (Illumina, USA) by RiboBio Biotechnology Co (Guangzhou, China). Read quality was examined using the FastQC software. Adapter removal and read trimming were performed by Trimmomatic (version 0.36). Paired-end reads were aligned to the mouse genome database (mm10) with HISAT2 software (version 2.0.5). HTSeq (version 0.12.4) was used to count the reads numbers mapped to each gene. Differential expression was assessed by DESeq2 in R [11]. The Benjamini–Hochberg multiple test correction method was enabled. Differentially expressed genes were chosen according to the criteria of fold change > 2 and adjusted P-value < 0.05.

### Bioinformatics analysis

Cluster software was used to perform hierarchical cluster analysis. Gene Ontology (GO) and Kyoto Encyclopedia of Genes and Genomes (KEGG) analyses were performed to determine the roles of the differentially expressed RNAs in Con-exo and HG-exo groups.

The potential biological functions of DElncRNAs enriched in the MAPK pathway were analyzed further. Pearson correlation coefficient (R) analysis was performed to evaluate co-expression of differentially expressed lncRNAs and mRNAs. A statistically significant difference was defined as P-value < 0.05 and R > 0.7. Motif information collected from JASPAR (http://jaspar.genereg.net) and HOCOMOCO [12] databases, and FIMO (Find Individual Motif Occurrence) software were used to predict transcription factors (TFs) of target sequences. Additionally the intersection of the results of the two databases was calculated to obtain candidate TFs. In accordance with the results, the lncRNA-TF-mRNA network was constructed by cytoscape software.

### Analysis of target LncRNA homology between mice and humans

The conservation of lncRNAs in humans and mice was evaluated by the phyloP (genetic p-value) conservation score. The conservation score of each locus of the screened lncRNAs was evaluated separately and the mean value of the conservation score was considered to be the conservation score of the region.

### Statistical analyses

All experiments were repeated three times. Data are presented as the mean ± standard deviation. Statistical analysis was carried out using SPSS 22.0 (IBM Corp., NY, USA). The t-test was used to evaluate differences between two groups. P-values of less than 0.05 were considered statistically significant.

## Results

### Characterization of exosomes

**Fig. 1:**
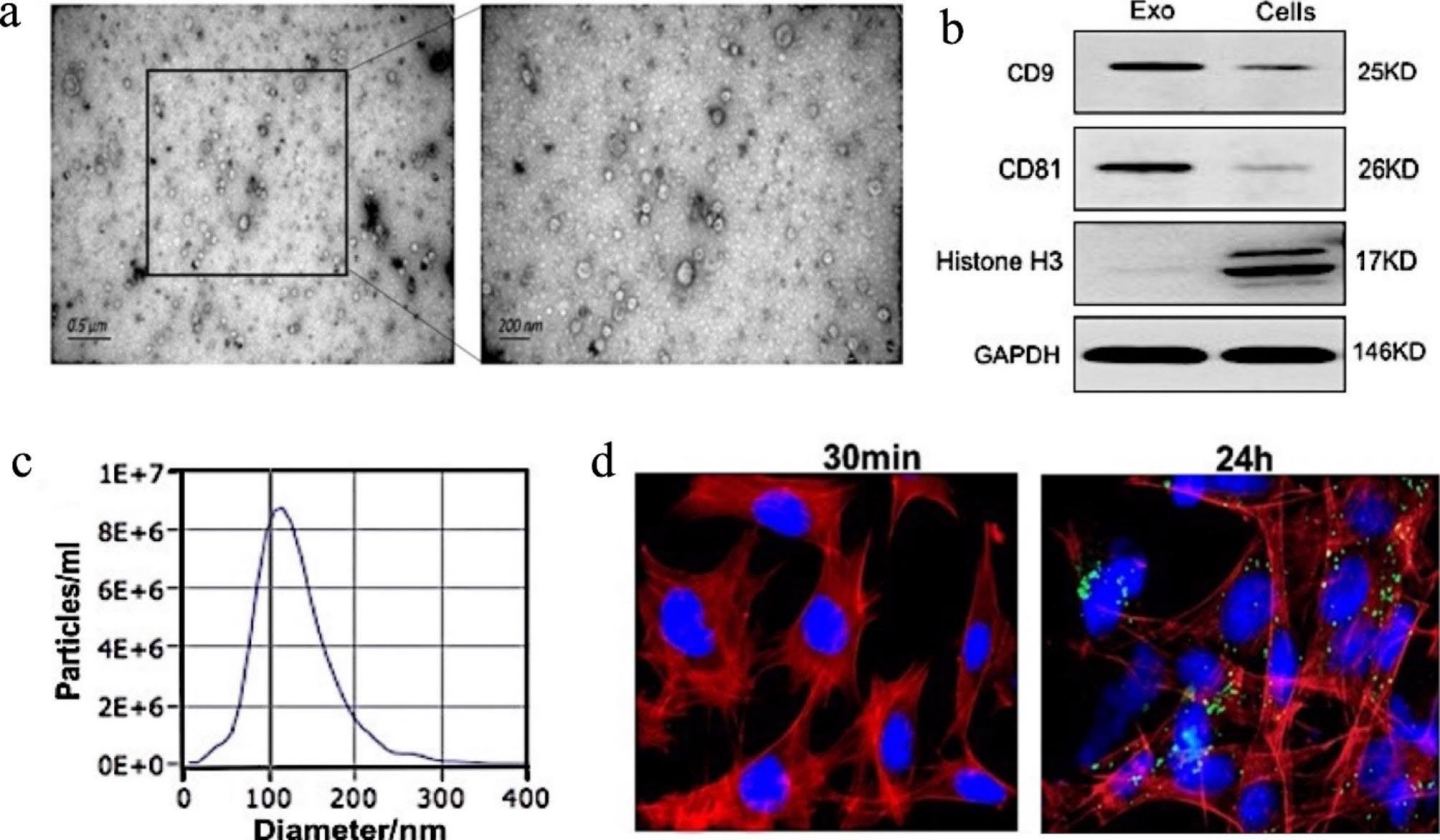
Characterization of macrophage-derived exosomes. Characterization of macrophage-derived exosomes. (**a**) Morphology of exosomes observed by transmission electron microscopy. Scale bars: 0.5 μm and 200 nm. (**b**) Exosome markers CD9 and CD81, nuclear marker histone H3 were detected in macrophage lysates and exosomes by western blotting. (**c**) Particle size and distribution analysis of exosomes. (**d**) Laser confocal microscopy of fluorescent dye-labeled exosomes and exosome uptake. Confocal microscopy showed uptake of PKH67-labeled (green) exosomes by mRTECs after treatment for 24 h (original magnification: ×400)

Electron microscopy showed double membrane-like structures with a circular shape (Fig. 1a). Next, western blotting showed that exosome markers CD9 and CD81 were highly expressed, while nuclear marker histone H3 was not expressed (Fig. 1b). The particles had diameters of 103.3 ± 8.9 nm as measured using ZETAVIEW (Fig. 1d). These findings confirmed that the exosomes extracted from macrophages were pure.

### MRTECs internalize macrophage-derived exosomes

We observed uptake of macrophage-derived exosomes by mRTECs by laser confocal microscopy. PKH fluorescent dye-labeled macrophage-derived exosomes were applied to mRTECs. After 30 min, no fluorescent dye was found around the nucleus of mRTECs. After 24 h, a large amount of PKH fluorescent dye was found in the cytoplasm of mRTECs (Fig. 1c). These results suggested that mRTECs effectively absorbed and internalized macrophage-derived exosomes.

### HG-exos induce mRTEC activation and EMT

**Fig. 2:**
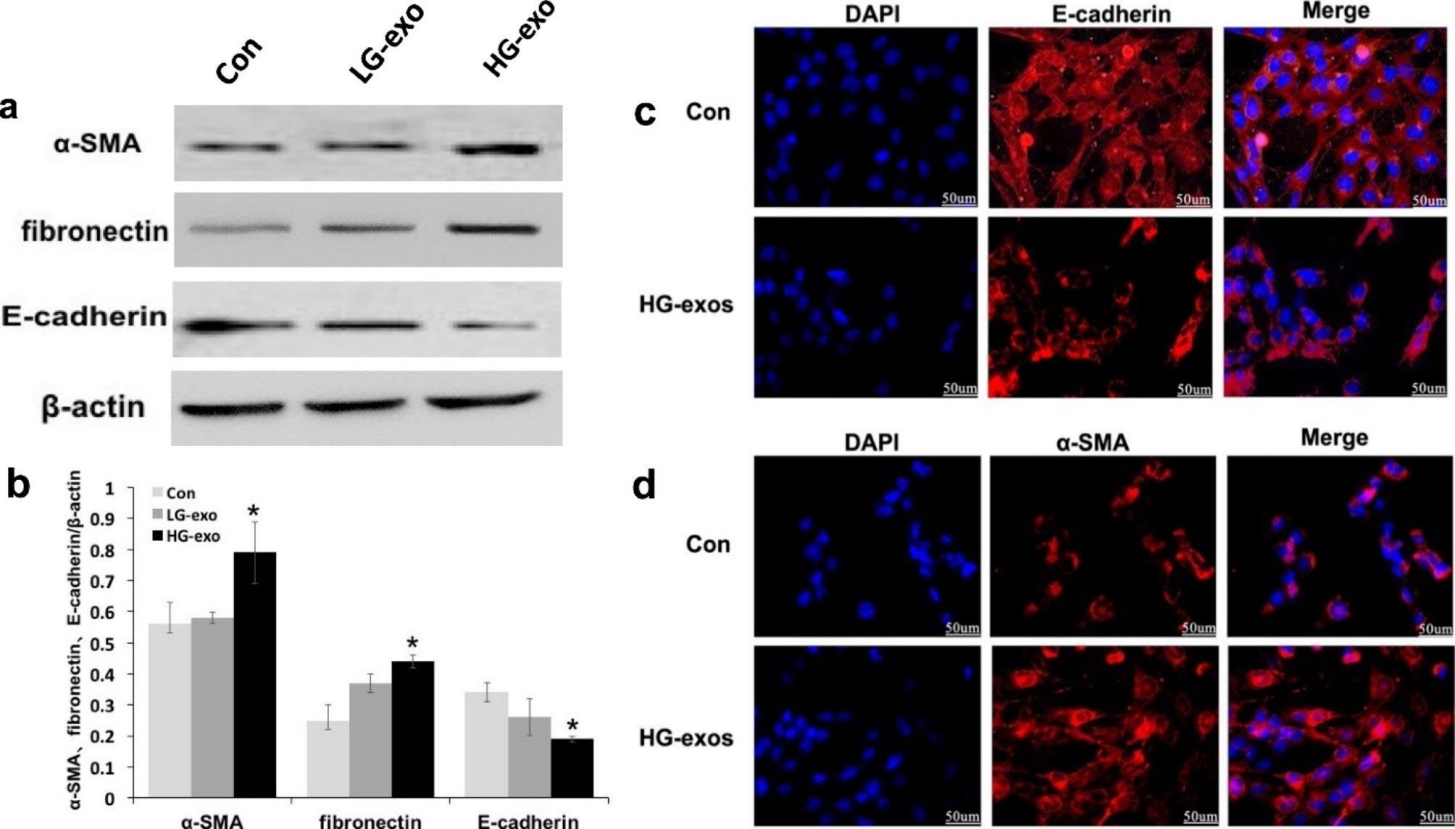
Effect of exosomes derived from HG-treated macrophages on tubular epithelial cells. (**a**) and (**b**) Expression of EMT-related markers, α-SMA, fibronectin, and E-cadherin detected by western blotting in mRTECs. (**c**) and (**d**) Representative fluorescence micrographs of mRTECs cocultured with various macrophage exosomes. Blue represented the nucleus and red represents the protein distribution. Data are presented as the mean ± SD, n = 3, *P < 0.05, compared with the Con group

**Fig. 3:**
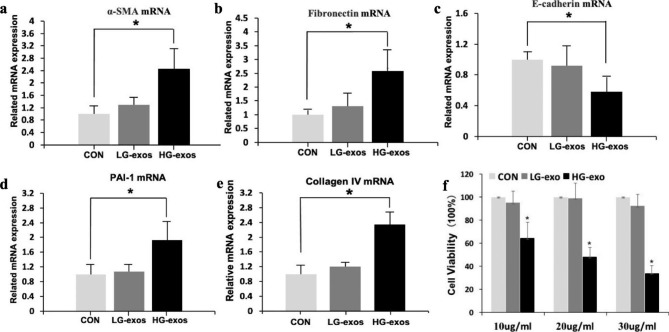
(**a**–**e**) Quantitative real-time polymerase chain reaction analysis of mRNAs associated with EMT and extracellular matrix components. (**f**) Proliferation of tubular epithelial cells assessed by the Cell Counting Kit-8 method

We detected the expression of EMT-related markers including α-SMA, fibronectin, and E-cadherin. Western blotting showed that HG-exos had markedly upregulated the expression of α-SMA and fibronectin compared with the control group (P < 0.05), but no significant difference was found compared with the LG-exo group (Fig. 2a and b). Moreover, E-cadherin expression was significantly deceased in the HG-exo group compared with the control group. Consistent with western blot analyses, RT-PCR showed that α-SMA and fibronectin mRNA levels were significantly higher, while the E-cadherin mRNA level was lower in the HG-exo group compared with the control group (Fig. 3a and c, P < 0.05). Immunofluorescence showed down-regulation of E-cadherin expression and upregulation of α-SMA expression in mRTECs treated with HG-exos (Fig. 2c and d). These data indicated that macrophage-derived exosomes induced a decrease in proliferation and promoted renal tubular EMT.

Next, mRTEC proliferation was assessed by the CCK-8 assay. As shown in Fig. 3f, mRTEC proliferation was significantly decreased to different various after the addition of various HG-exo concentrations compared with the control group (P < 0.05). The inhibitory effect was more obvious at high exosome concentrations.

### HG-exos induce extracellular matrix overproduction in MRTECs

To confirm that HG-exos induced extracellular matrix overproduction in mRTECs, we examined gene expression levels of some extracellular matrix-related markers. RT-PCR was performed to detect Collagen IV and Plasminogen activator inhibitor 1 (PAI-1) mRNA expression. As a result, Collagen IV and PAI-1 expression levels were significantly upregulated in the HG-exo groups compared with the control group (Fig. 3d and e). These data suggested that macrophages mediated extracellular matrix deposition of renal tubular epithelial cells through exosomes.

### Bioinformatics analyses of gene expression

**Fig. 4:**
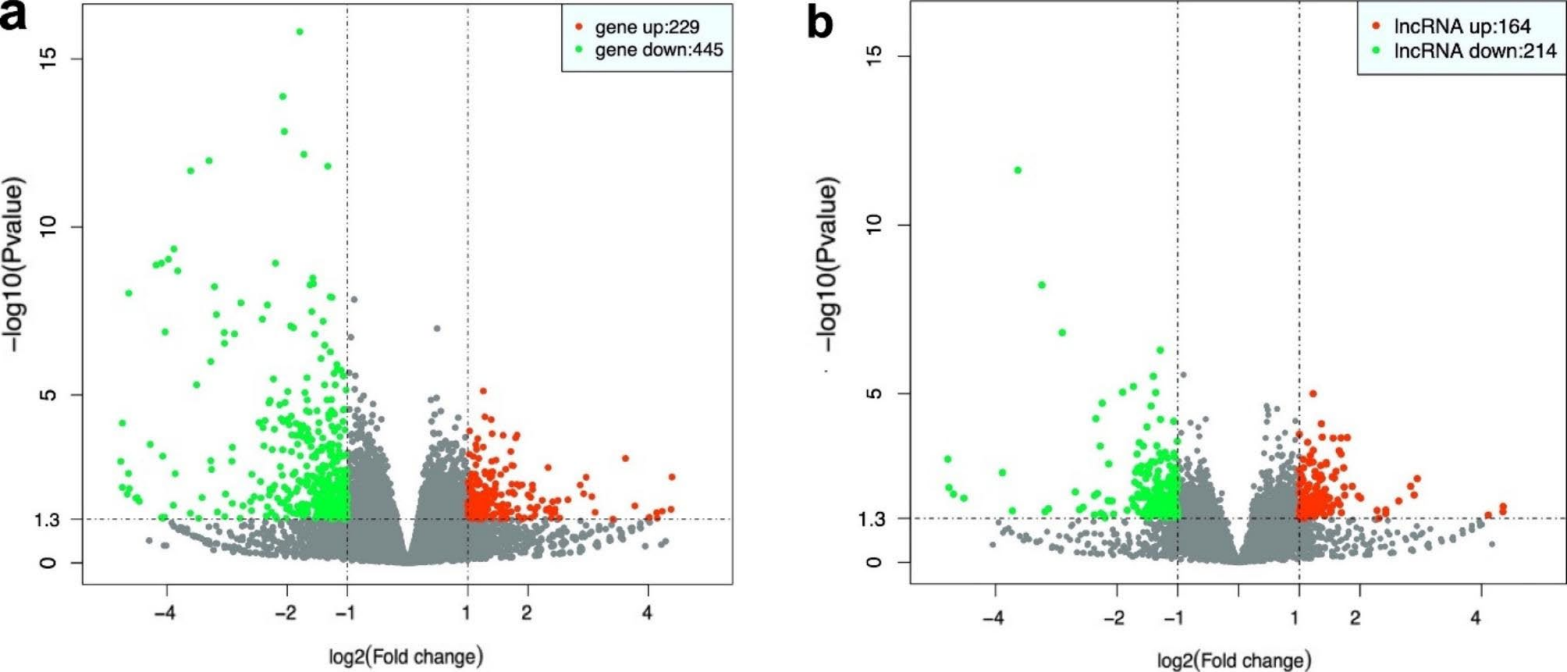
Volcano map of differentially expressed mRNAs (**a**) and lncRNAs (**b**) respectively. Horizontal and vertical axes in the figure represent Log_2_ (fold change) and Log_10_ (p-value)

**Fig. 5:**
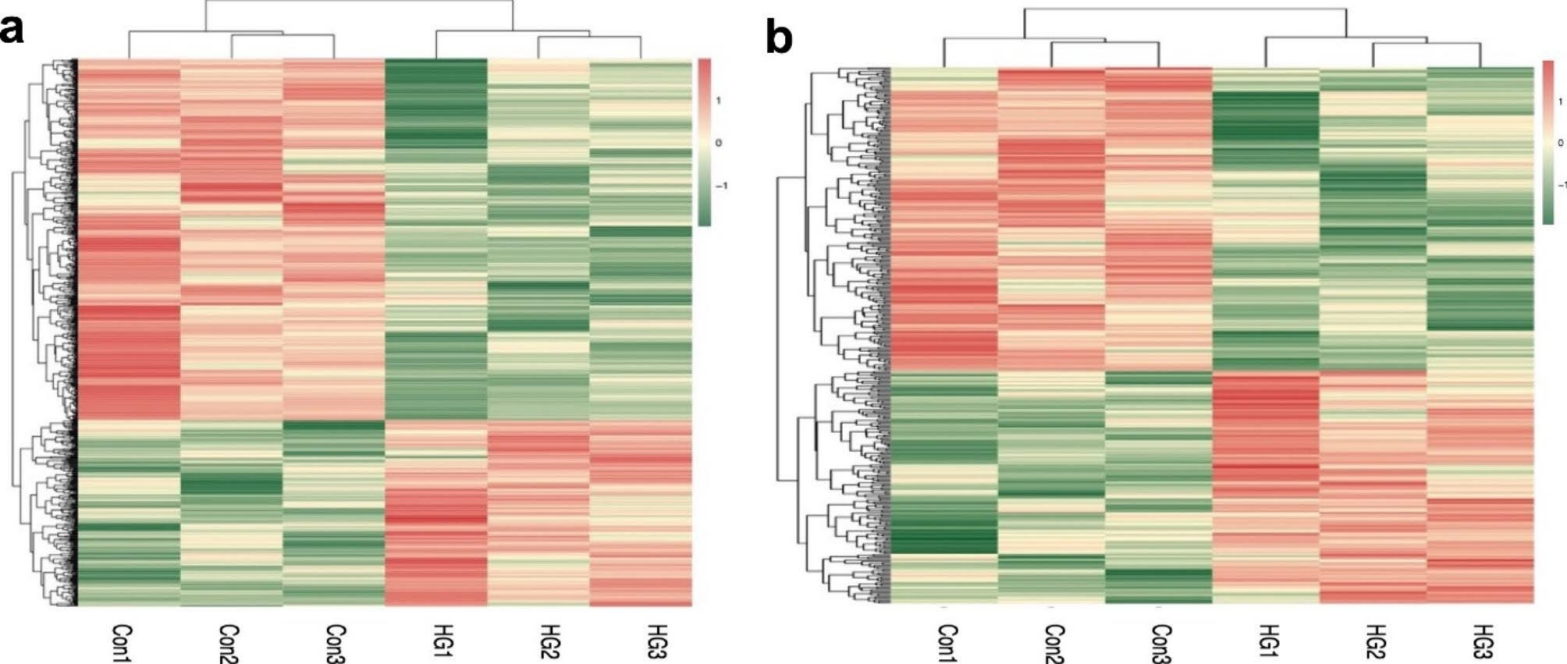
Cluster analysis of differentially expressed mRNAs (**a**) and lncRNAs (**b**) respectively

**Fig. 6:**
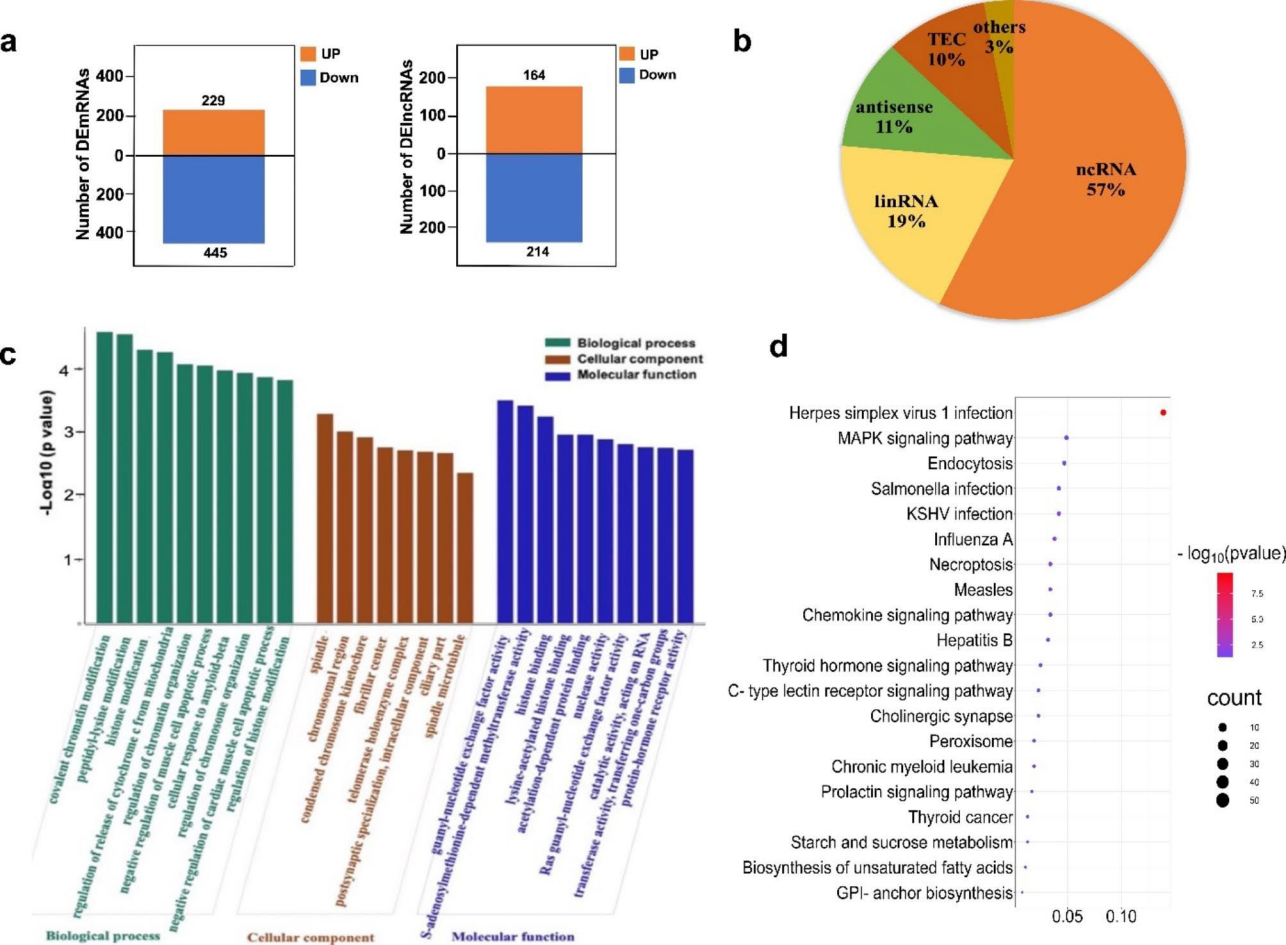
Bioinformatics analysis of lncRNAs. (**a**) Numbers of DEmRNAs and DElncRNAs. (**b**) Pie chart of different lncRNA types. Orange: non-coding RNA (ncRNA); Yellow: long interspersed ncRNA (lincRNA); Green: antisense lncRNA; Red: to be experimentally confirmed (TEC); Khaki: other lncRNA kinds. (**c**) GO enrichment analysis revealed biological processes, cellular components, and molecular functions. (**d**) Functional KEGG pathway enrichment analysis using DAVID (www.kegg.jp/kegg/kegg1.html). The top 20 enrichment pathways are shown in the bar chart

A volcano plot (Fig. 4) and cluster analysis (Fig. 5) showed significant DElncRNA distribution. There were 674 DEmRNAs in HG-exos, of which 229 were upregulated and 445 were downregulated. Additionally, 378 lncRNAs were significantly differentially expressed in HG-exos, of which 164 were upregulated and 214 were downregulated (Fig. 6a).

DElncRNAs were divided into the following categories in accordance with their predictive functions: 57% non-lncRNAs, 19% long interspersed ncRNAs, 11% antisense lncRNAs, 10% to be experimentally confirmed, and approximately 3% other kinds of lncRNAs (Fig. 6b).

The top GO terms associated with biological processes, cellular components, and molecular functions are presented in Fig. 6c (P < 0.05). In the biological processes, the top five GO terms were peptidyl-lysine modification, histone modification, regulation of release of cytochrome c from mitochondria, regulation of chromatin organization, and negative regulation of the muscle cell apoptotic process. In the cellular components, the top five GO terms were spindle, chromosomal region, condensed chromosome kinetochore, fibrillar center, the and telomerase holoenzyme complex. In the molecular functions, the top five GO terms were guanyl–nucleotide exchange factor activity, S-adenosylmethionine-dependent methyltransferase activity, histone binding, lysine-acetylated histone binding, and acetylation-dependent protein binding.

KEGG analysis revealed that the differentially expressed genes were significantly involved in the Chemokine signaling pathway, Prolactin signaling pathway, MAPK signaling pathway, Peroxisome, C-type lectin receptor signaling pathway, endocytosis, thyroid hormone signaling pathway, necroptosis, and thyroid cancer (Fig. 6d).

### LncRNA-TF–mRNA network analysis

**Fig. 7:**
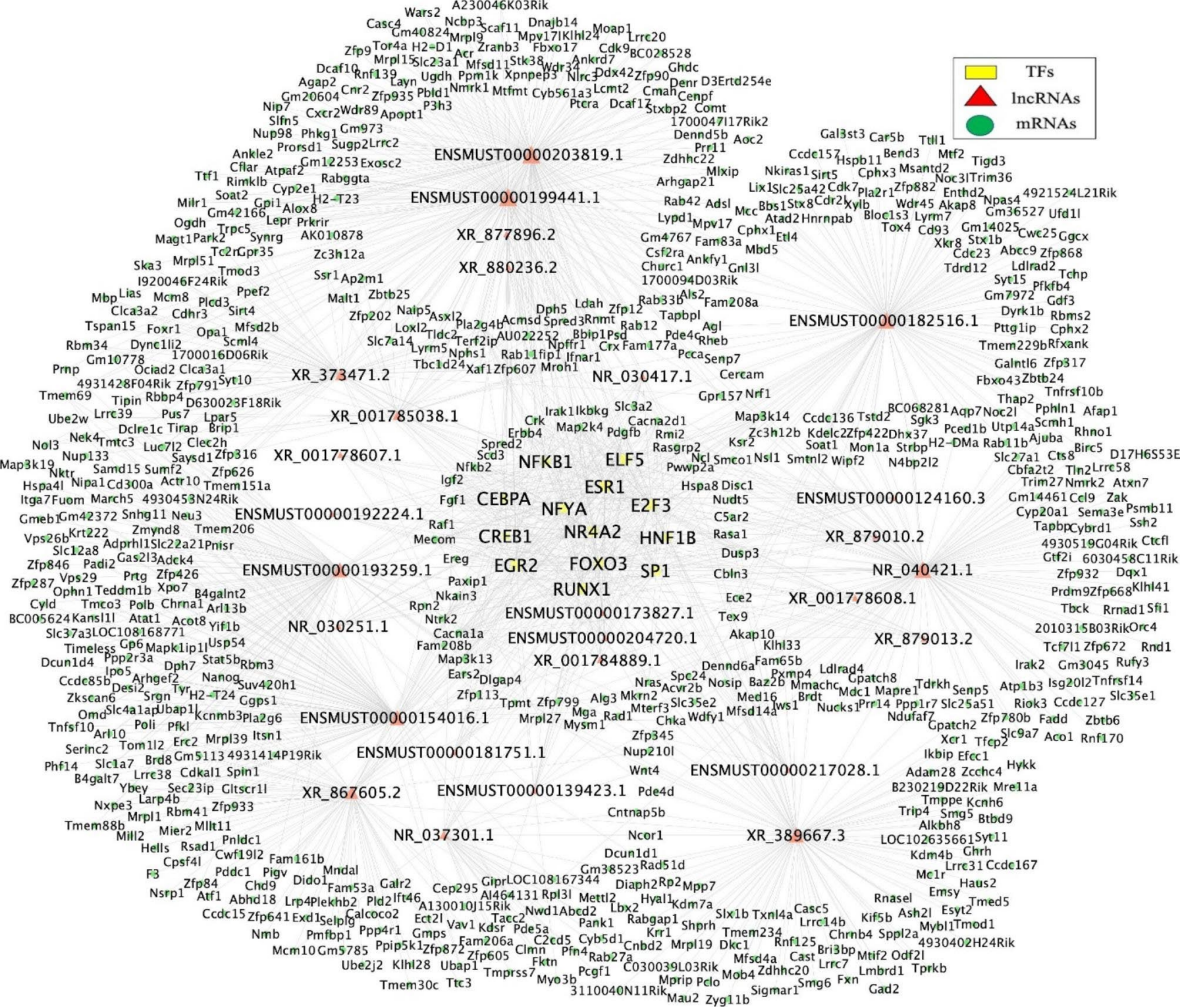
LncRNA–TF–mRNA network analysis. There were 13 TFs, 27 lncRNAs, and 677 mRNAs in the network. Yellow nodes represent TFs, red nodes represent lncRNAs, Green nodes represent target genes, and the node size is proportional to its outward connection. LncRNA: long non-coding RNA; TF: transcription factor

Next, we focused on the biological functions of lncRNAs enriched in the MAPK pathway To this end, we constructed a lncRNA-TF–mRNA regulatory network (Fig. 7). In this network, 13 TFs, 27 lncRNAs, and 677 mRNAs were included. In HG-exos, some TFs, including HNF1 homeobox B (HNF1B), cAMP responsive element binding protein 1 (CREB1), specificity protein 1 (SP1), Runt-related transcription factor 1 (RUNX1), and E74-like factor 5 (ELF5) have been verified to be closely related to EMT.

### Homology analysis and QPCR validation

**Fig. 8:**
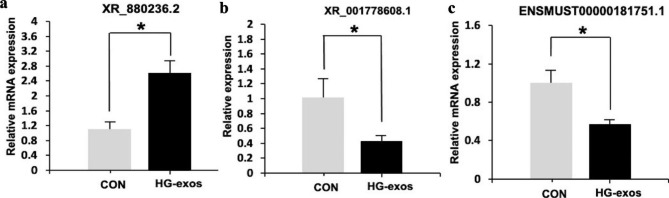
Verification of differential lncRNA expression by RT-qPCR. The expression levels of individual lncRNAs were compared between Con and HG-exo groups. Data are the mean ± SD, *P < 0.05

Some lncRNAs enriched in the MAPK signaling pathway may be involved in EMT. Therefore, we performed homology analysis of target lncRNAs between mice and humans. The most conserved lncRNAs enriched in the MAPK pathway were ENSMUST00000181751.1, XR_001778608.1, and XR_880236.2, which had conservation scores of > 0.70. The specific information of these lncRNAs showed in Table 2.The expression of these lncRNAs was verified in control and HG-exo groups by qPCR. As shown in Fig. 8, XR_880236.2 expression was upregulated significantly, while the other lncRNAs (ENSMUST00000181751.1 and XR_001778608.1) showed significant downregulation in the HG-exo group compared with the control group.

**Table 2:** Information of target transcripts

LncRNA transcript name	Gene name	Coding potential	Log_2_(fold_change)	Chromosomal location
ENSMUST00000181751.1	ENSMUSG00000097554.1	non-coding	-1.36	chr5
XR_001778608.1	Gm39212	non-coding	-1.21	chr8
XR_880236.2	Gm39396	non-coding	1.32	chr11

## Discussion

EMT is characterized by loss of epithelial markers, acquisition of mesenchymal markers, cytoskeleton remodeling, and finally conversion to myofibroblasts [13]. It has been reported that 36% of myofibroblasts originate from transdifferentiation of tubular epithelial cells in kidney fibrosis [14]. Therefore, EMT is considered to be a critical step of kidney fibrosis.

Macrophages play a major role in RIF pathogenesis [15, 16]. In the UUO model, RIF severity reduces significantly after specific deletion of macrophages [17]. Thus, macrophages may be a potential target to prevent or treat DN. In our study, we isolated and verified exosomes secreted from macrophages stimulated by high glucose. PKH67-labeled exosomes were dramatically internalized by tubular epithelial cells. A previous study reported that exosomes derived from macrophages are taken up by mesangial cells under hyperglycemia [18]. Therefore, we determined the biological function of this kind of exosome in DN.

We observed that HG-exos significantly inhibited the proliferation of tubular epithelial cells. With the increase in exosome concentration, proliferation was decreased further. Additionally, exosomes markedly deceased E-cadherin expression and enhanced α-SMA and fibronectin expression, which strongly suggested that HG-exos induced EMT of mRTECs. The expression levels of type IV collagen and PAI-1, which are ECM markers, were up-regulated after 72 h of treatment. Therefore, it is reasonable to speculate that these exosomes serve as external stimuli for tubular epithelial cells by modulation of the microenvironment.

LncRNAs are expected to become sensitive diagnostic indicators and have therapeutic applications for various diseases [19]. We carried out high-throughput sequencing of exosomes to understand the biological function of exosomal lncRNAs. In total, 378 lncRNAs were significantly differentially expressed between control and HG-exo groups, of which 164 were upregulated and 214 were downregulated. RT-qPCR supported the reliability of these findings, suggesting that exosomal lncRNAs are related to EMT induction in mRTECs.

A functional lncRNA–mRNA regulatory network was constructed. The lncRNAs were associated with multiple KEGG pathways among which Chemokine signaling and MAPK signaling pathways have been widely reported to be associated with EMT [20]. Therefore, lncRNAs in HG-exos are likely to affect important EMT-related pathways.

LncRNAs participate in intercellular signaling through various mechanisms. First, lncRNAs act as modulators of signaling pathways under various stimuli and participate in specific signal transduction processes. Another mechanism of lncRNAs is represented as a molecular decoy model. For example, lncRNA can act as an miRNA sponge to interfere with the function of miRNAs and further disrupt target genes downstream [21]. LncRNA-mediated transcriptional regulation can affect transcription, mRNA stability, or translation [22]. Recent studies have implied aberrantly expressed lncRNA in DN patients, suggesting that lncRNAs play a role in the mechanisms of EMT and kidney fibrosis [23, 24]. LncRNA maternally expressed gene 3 is upregulated in the serum of diabetic patients. Importantly, knockdown of this lncRNA reduces the expression of type IV collagen and fibronectin in kidney tissues, indicating that this lncRNA has a pathological effect on DN [25]. Nuclear-enriched transcription-1 is another lncRNA involved in DN progression, which targets microRNA to promote extracellular matrix accumulation and EMT via the Akt/mTOR signaling pathway [26]. Our study revealed some novel lncRNAs that may be associated with EMT.

Network analysis showed that lncRNAs enriched in MAPK signaling may regulate several EMT-related TFs including CREB, Sp1, RUNX1, ELF5, and HNF1B. Transcription factor CREB is involved in inflammation, oxidative stress, and cellular uptake of uremic toxins. A study of osteosarcoma found that CREB promotes EMT by activating the PI3K/AKT/mTOR pathway [27]. Additionally, transcription factor Sp1 binds to the promoter of the E-cadherin-encoding gene and crucially affects its expression [28]. RUNX1 belongs to the Runx family that to interacts with Smads as coactivators. A recently study has verified that knockdown of RUNX1 attenuates TGF-β-induced EMT of tubular epithelial cells, indicating that RUNX1 may be a potential target to prevent renal fibrosis [29]. Our study revealed that some lncRNAs are associated with the abovementioned TFs and that these TFs may be involved in EMT induction.

To extend this study to DN patients, we performed homology analysis of lncRNAs. The most conserved lncRNAs were ENSMUST00000181751.1, XR_001778608.1, and XR_880236.2, which has conservation scores higher than 70%. Thus, the bioinformatics mining has laid a solid foundation for mechanistic research and the exact roles of these lncRNAs remain to be determined.

## Conclusion

We found that exosomes from HG-treated macrophages induce mRTEC activation increase phenotypic conversion marker expression. Additionally, lncRNA expression profiles in HG-treated macrophage exosomes may provide potential information to further explore the roles of macrophages in the pathogenesis of tubular EMT.

## Electronic supplementary material

Below is the link to the electronic supplementary material.

Supplementary Material 1

## Data Availability

The datasets generated during the current study are available in the SRA repository, ACCESSION NUMBER: PRJNA872872. https://dataview.ncbi.nlm.nih.gov/objects?linked_to_id=SRR21227974&archive=bioproject

## List of abbreviations

DN: diabetic nephropathy
RIF: renal interstitial fibrosis
EMT: epithelial–mesenchymal transition
lncRNAs: long non-coding RNAs
HG-exos: high glucose-treated macrophage exosomes
mRTECs: mouse renal tubular epithelial cells
OD: optical density
DEmRNAs: differentially expressed mRNAs
DElncRNAs: differentially expressed lncRNAs
FC: fold-change
GO: Gene Ontology
KEGG: Kyoto Encyclopedia of Genes and Genomes
TFs: transcription factors
PAI-1: plasminogen activator inhibitor 1
HNF1B: HNF1 homeobox B
CREB1: cAMP responsive element binding protein 1
SP1: specificity protein 1
RUNX1: runt-related transcription factor 1
ELF5: E74-like factor 5
