# Supplementary figures and images for: Exosomes from high glucose-treated macrophages promote epithelial–mesenchymal transition of renal tubular epithelial cells via long non-coding RNAs
Source: BMC Nephrol. 2023 Jan 30;24:24. doi: 10.1186/s12882-023-03065-w (PMC9887774; doi:10.1186/s12882-023-03065-w)

## Slide 1
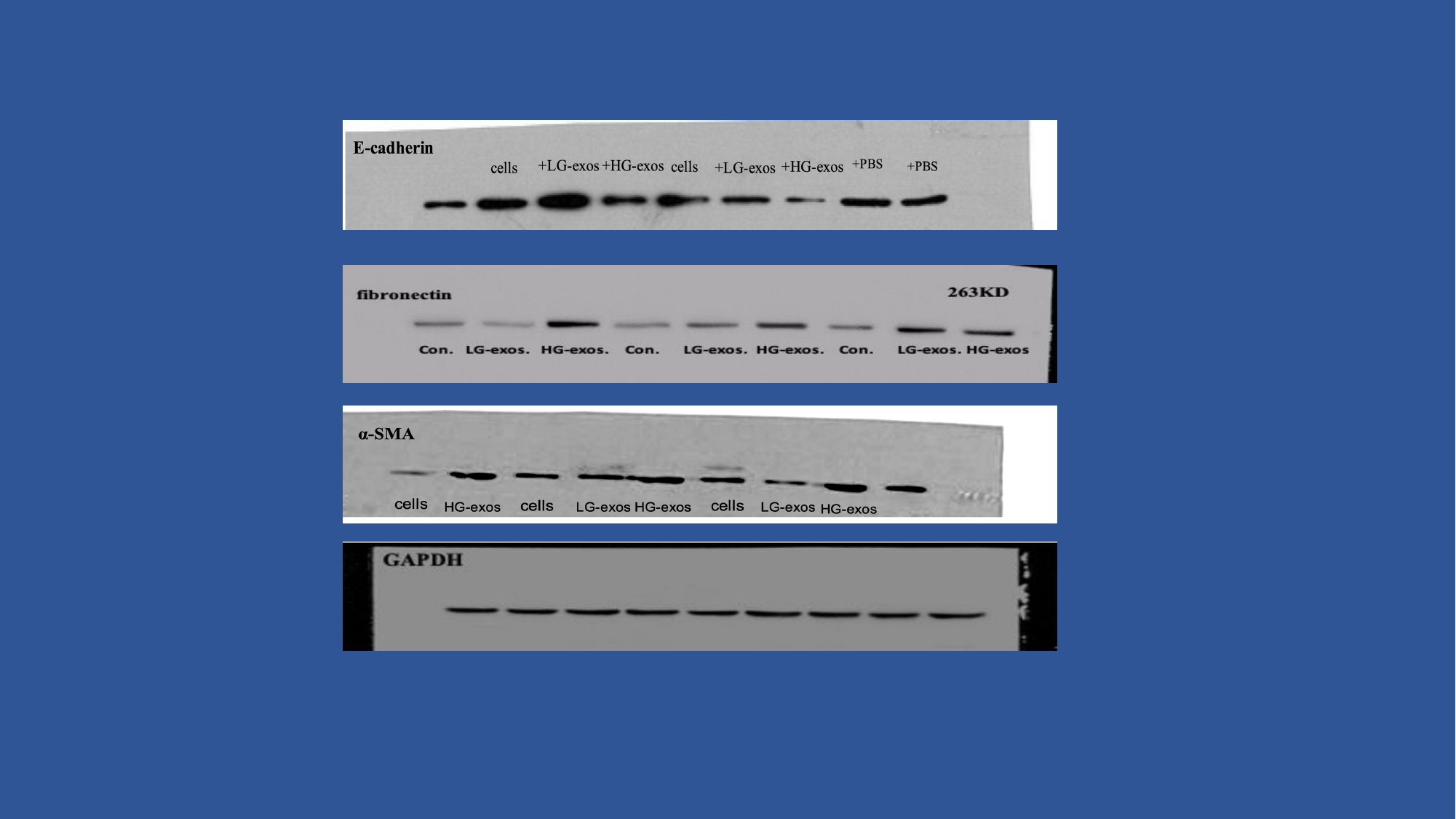

## Slide 2
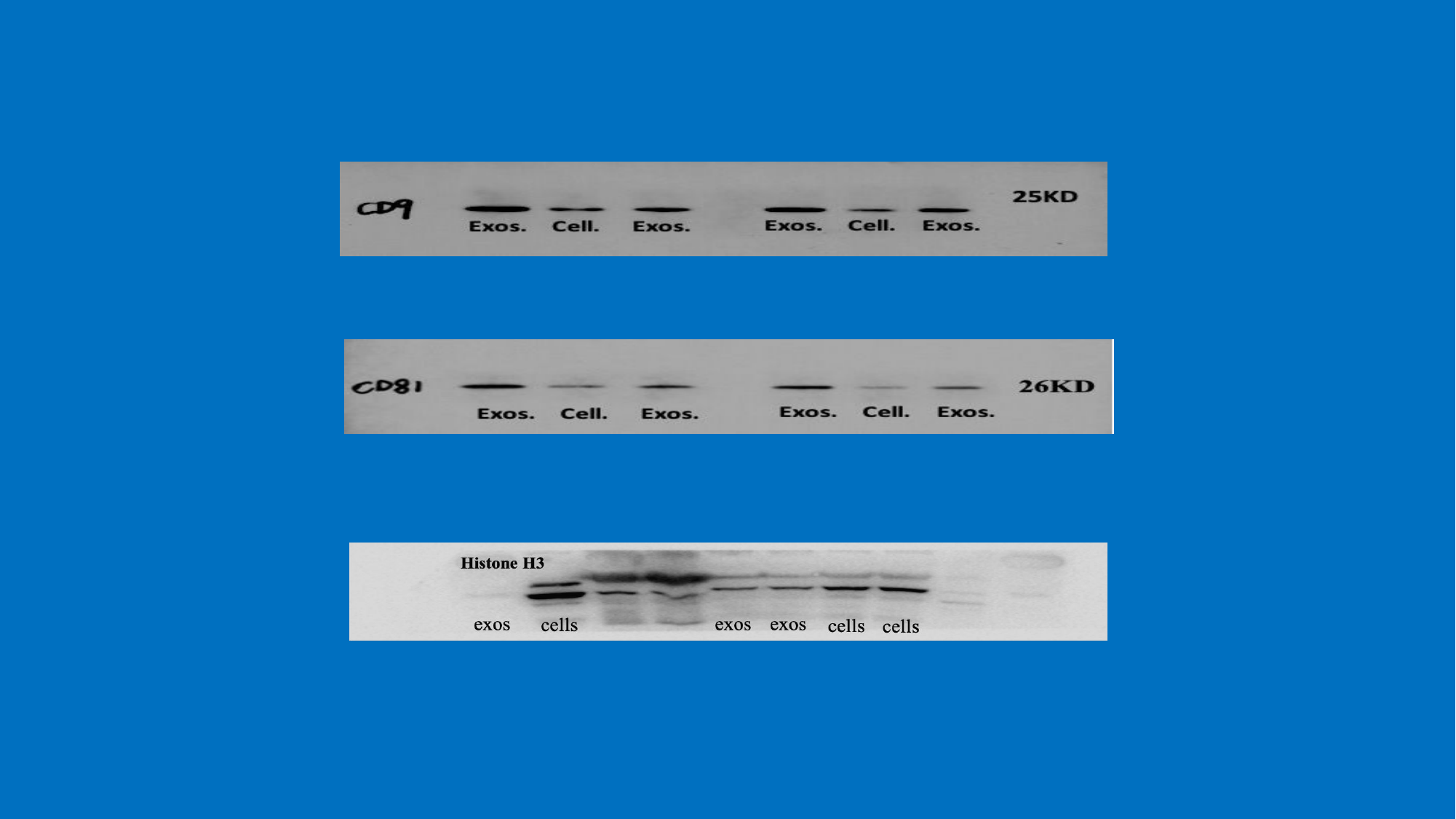

Supplement: Supplementary file 1 — Supplementary Material 1 [file 12882_2023_3065_MOESM1_ESM.pptx]
